# Health changes during Covid-19: a nationwide study with dental students

**DOI:** 10.11606/s1518-8787.2023057004666

**Published:** 2023-03-30

**Authors:** Ane Poly, Laísa Inara Gracindo Lopes, João Victor Frazão Câmara, Suelem Chasse Barreto, Gisele Damiana da Silveira Pereira

**Affiliations:** I Universidade Federal do Rio de Janeiro Faculdade de Odontologia Departamento de Odontoclínica Rio de Janeiro RJ Brasil Universidade Federal do Rio de Janeiro. Faculdade de Odontologia. Departamento de Odontoclínica. Rio de Janeiro, RJ, Brasil; II Saarland University University Hospital Clinic of Operative Dentistry, Periodontology and Preventive Dentistry Homburg Saar Germany Saarland University. University Hospital. Clinic of Operative Dentistry, Periodontology and Preventive Dentistry. Homburg/Saar, Germany

**Keywords:** Students, Dental, Adaptation, Psychological, Health Behavior, Physical Distancing, COVID-19

## Abstract

**OBJECTIVE:**

To assess the changes in stress levels, social behavior, dietary and parafunctional habits, oral hygiene, among other conditions perceived by dental students in Brazil during the Covid-19 pandemic and evaluated the correlations between stress level and other variables.

**METHODS:**

An online questionnaire was developed and validated. Undergraduates enrolled in private and public dental schools were recruited by convenience sampling. Data were collected on the perceived changes regarding stress levels, financial and social characteristics, dietary habits, oral hygiene, health conditions, and parafunctional habits. Quantitative variables were expressed as absolute and relative frequencies. Wilcoxon test evaluated comparisons between perceived changes, and correlations between changes in stress levels and other variables were analyzed by Spearman correlation (α = 0.05).

**RESULTS:**

A total of 638 dental students, mean age of 22.95 ± 4.10 years, participated in the study. During the pandemic, the reported stress levels increased while household income decreased (p < 0.05). Late dinners and mindless eating increased in frequency, whereas oral hygiene decreased (p < 0.05). Most of the health conditions and parafunctional habits assessed changed (p < 0.05). Perceived stress levels showed poor negative correlations with household income (r_S_ = −0.14), poor positive correlations with the pressure to contribute financially in the household (r_S_ = 0.19), and poor positive correlations with food choice frequency (r_S_ = 0.15) (p < 0.05).

**CONCLUSIONS:**

Dental students reported perceived changes in stress levels, dietary habits, oral hygiene, health conditions, parafunctional habits, and social behavior. Moreover, the results showed poor correlations, as students with higher stress levels tended to have the lowest household income, feel pressured to contribute financially in the household, and present a high meal intake frequency.

## INTRODUCTION

Historically, several infectious disease outbreaks have taken place around the world, bringing significant consequences for humanity^1^. In 2019, news reported the first cases of Covid-19, caused by the new SARS-CoV-2 virus^2^. Due to its severity and rapid contagion spread, the World Health Organization (WHO) declared Covid-19 an international public health emergency and a pandemic^3,4^.

Its fast transmission rate, relevant medical complications, and high associated morbidity led to the proposition of several sanitary measures by infectiologists and world health authorities. Among those put in place to stop Covid-19 dissemination, social distancing proved the most challenging^3,5^.

A large portion of the world population went into quarantine, sequestered in their homes, which led to strict adaptations of daily habits and exacerbated changes in social dynamics^6^. Studies have shown that psychological disorders caused by social distancing, regardless of socioeconomic class, highly affect youth, known to have a greater need for socialization^3,7^.

The risk of imminent contamination, the uncertainty of its duration, the consequent financial losses, and several other factors related to the Covid-19 pandemic caused natural psychological responses like depression, anxiety, stress, and panic disorders to situations of sudden random changes^7^. Such negative emotions can lead to immunosuppression, which has deleterious health consequences^8^.

Moreover, psychological factors influence muscle hyperactivity, resulting in temporomandibular disorders and parafunctional activities such as clenching and bruxism^9^. Behavioral changes can also trigger eating disorders, alcoholism, changes in sleep rhythm, among other conditions that culminate in oral health problems^10^. Besides, studies show that the rates of inadequate, compulsive, or less healthy dietary habits increased throughout the pandemic^11^.

The severity of Covid-19 presented clear challenges for dentistry, considering the high chances of contamination due to patient proximity and the aerosols generated during clinical procedures^12^. Consequently, dental schools in Brazil had to suspend their in-person classes and training, thus implementing an emergency remote education system until the schools could reopen safely. Dental education is a demanding field and relies greatly on on-site instruction and hands-on practice for acquiring the necessary clinical skills^13,14^. In this pandemic scenario, dental students have been particularly affected by anxiety associated with learning complex clinical procedures and managing busy and challenging academic schedules^15,16^.

Other countries have investigated the psychological and financial impact of the Covid-19 pandemic on dental students^17,18^. A recent study conducted with Brazilian dental undergraduates found a high prevalence of alcohol abuse and anxiety during the pandemic^19^. But further information on behavioral and health changes is still missing. Thus, this study sought to assess the perceived changes in stress levels, dietary habits, oral hygiene, health conditions, parafunctional habits, and social behavior during the Covid-19 pandemic among Brazilian dental undergraduates. A second objective was to correlate stress levels with the other variables evaluated. Our null hypothesis posited that no perceived changes and no correlations regarding all variables would be found.

## METHODS

We designed a nationwide quantitative, observational, cross-sectional survey, which was approved by the local Ethics Committee and registered in the Open Science Framework (OSF) database (doi: 10.17605/OSF.IO/48YUG). This study complied with all provisions of the local oversight committee guidelines on human subjects and HUCFF-UFRJ policies (protocol 4.422.128). This report followed the Survey Reporting Guideline (SURGE)^20^.

### Sample Calculation and Participant Selection

Considering the 600936 students enrolled in 553 dental schools in Brazil^21^, we calculated the sample size at a 50% accuracy, 95% confidence interval and a 4% margin of error, using G*Power 3.1.9.2 software (Heinrich Heine, Universität Düsseldorf, Dusseldorf, Germany), resulting in a minimum survey respondent sample of 600 participants.

Based on a convenience sampling method, we emailed students enrolled in private or public dental schools from all five regions of Brazil (North, Northeast, Midwest, Southeast, and South). Eligibility criteria consisted of people of all ages, genders, and socioeconomic statuses. Only participants who failed to provide their written informed consent were excluded from the study.

### Questionnaire Description

We developed an initial set of items based on seven domains: sociodemographic characteristics (program year, region, gender, age and household income); perceived stress levels; dietary behaviors (meals and food choices); oral hygiene; health conditions (gingivitis, tooth wear, pains, ulcers and herpes); parafunctional habits (clenching, grinding and nail, object, finger and lip biting); and social behaviors (sleep hours and alcohol consumption).

Paired experts from the following seven fields of knowledge analyzed the items for possible flaws and suggestions: social work, cariology, temporomandibular dysfunction, stomatology, nutrition, periodontics, and psychology. We calculated the agreement rate (%) between experts for each domain. Each item was individually validated by calculating the Content Validity Index (CIV). Expert agreement rate was 100% for all domains. CIV was 1 for all items, except for items 9, 10, 13, and 14, which had a CIV of 0.5. After adjustments and a new evaluation, these items reached a CIV of 1.

Subsequently, we performed a pretest with 32 randomly selected dental students. They were asked to determine item intelligibility and make relevant suggestions. Items 8, 9, 10, 11, 12, 22, 23, and 24 were adjusted based on their answers, after which the questionnaire was considered validated and ready to be applied.

The 27-item questionnaire was uploaded to Google Forms (Google LLC, Mountain View, USA). Its first section (questions 1 to 5) included demographic and social characteristics; whereas the second section consisted of variables associated with the pandemic (questions 6 to 27). Five variables included a five-point scoring system: Monthly household income (questions 6 and 7), ranging from 1 (“1 - 4 minimum wages”) to 5 (“20+ minimum wages”); Stress levels (questions 9 and 10), ranging from 1 (“not stressed”) to 5 (“extremely stressed”); Food choice frequency (questions 15 and 16), ranging from 1 (“Never”) to 5 (“7+ times a week”); Tooth brushing frequency (questions 18 and 19), ranging from 1 (“No brushing”) to 5 (“4+ a day”); Tooth brushing time (questions 20 and 21), ranging from 1 (“After meals and after snacks”) to 5 (“Never”).

Responses were collected from January to April 2021 and tabulated.

### Statistical Analysis

Data collected via the online questionnaire were analyzed using BioEstat 5.3 (BioEstat, Belém, Brazil). Descriptive analyses were expressed in absolute and relative frequencies (%). As data collected on stress levels failed to meet the threshold for normality (Shapiro-Wilk test; p < 0.05), we adopted nonparametric methods. Within-subjects comparison between participants’ perceptions before (past self-report) and during the pandemic for the ordinal variables (monthly household income, stress levels, food choice frequency, tooth brushing frequency and tooth brushing time) was calculated by Wilcoxon test. Within-subjects comparison between participants’ perceptions before (past self-report) and during the pandemic for the categorical variables (meals, food choice, health conditions and parafunctional habits) was estimated by McNemar test. Spearman correlation was used to analyze correlations between changes in stress levels and the other variables. Significance level was set at 5%.

## RESULTS

A total of 638 dental undergraduates answered the survey, of which 48.4% were fourth- and fifth-year students, whereas 51.6% were approaching graduation. Regarding dental school, 23.1% of the participants were enrolled in private institutions and 76.9% in public dental schools. Age ranged from 17 to 67 years old, with mean age of 22.95 ± 4.10 years. Table 1 summarizes the distribution of respondents according to sociodemographic and educational characteristics.

**Table 1:** Educational and sociodemographic characteristics of study participants.

Variable	n	%
1. Dental school year		
1st year	101	15.8
2nd year	139	21.8
3rd year	148	23.2
4th year	162	25.4
5th year	88	13.8
2. Dental school region		
North	14	2.2
Northeast	157	24.6
Midwest	22	3.5
Southeast	403	63.1
South	42	6.6
3. Gender		
Female	514	80.6
Male	124	19.4
4. Age (years)		
< 20	57	8.9
20–25	491	76.9
26–30	71	11.1
> 30	19	3.1
5. Main income shared by		
1 person	45	7.1
2–3 persons	259	40.6
4–5 persons	298	46.7
> 5 persons	36	5.6

Table 2 presents the absolute and relative frequencies (%) of perceived changes in variables associated with the pandemic. Up to 69.8% of the students admitted feeling pressured to financially contribute in their household, showing a poor correlation to senior students (r_S_ = 0.14, p = 0.0006) and a fair correlation to students living with reduced household income (r_S_ = −0.28, p < 0.0001). Moreover, feeling pressured to contribute financially showed a poor correlation to higher stress levels (r_S_ = 0.19, p < 0.0001).

**Table 2:** Absolute and relative frequencies (%) of participants’ perception of variables associated with the pandemic.

Variable	n	%
6. Monthly household income BEFORE the pandemic (consider a minimum wage of R$1,045):		
(1) 1–4 minimum wages	307	48.1
(2) 5–9 minimum wages	195	30.6
(3) 10–14 minimum wages	93	14.6
(4) 15–19 minimum wages	25	3.9
(5) 20+ minimum wages	18	2.8
7. Monthly household income DURING the pandemic (consider a minimum wage of R$1,045):		
(1) 1–4 minimum wages	348	54.6
(2) 5–9 minimum wages	181	28.4
(3) 10–14 minimum wages	69	10.8
(4) 15–19 minimum wages	27	4.2
(5) 20+ minimum wages	13	2.0
8. Do you feel pressured to help your family financially due to the pandemic?		
Yes, my family pressures me, and I pressure myself	60	9.4
Yes, my family pressures me, but I do not pressure myself	1	0.2
Yes, I pressure myself, but my family does not pressure me	385	60.3
No	192	30.1
9. Stress levels BEFORE the pandemic:		
(1) not stressed	31	4.9
(2) a little stressed	263	41.2
(3) moderately stressed	262	41.1
(4) very stressed	67	10.5
(5) extremely stressed	15	2.3
10. Stress levels DURING the pandemic:		
(1) not stressed	3	0.5
(2) a little stressed	34	5.3
(3) moderately stressed	171	26.8
(4) very stressed	256	40.1
(5) extremely stressed	174	27.3
11. Meals eaten BEFORE the pandemic:		
Breakfast		
Yes	481	75.4
No	157	24.6
Morning snack		
Yes	167	26.2
No	471	73.8
Lunch		
Yes	622	97.5
No	16	2.5
Afternoon snack		
Yes	445	69.7
No	193	30.3
Dinner		
Yes	581	91.1
No	57	8.9
Late dinner		
Yes	120	18.8
No	518	81.2
Mindless eating		
Yes	224	35.1
No	414	64.9
12. Meals eaten DURING the pandemic:		
Breakfast		
Yes	451	70.7
No	187	29.3
Morning snack		
Yes	154	24.1
No	484	75.9
Lunch		
Yes	611	95.8
No	29	4.2
Afternoon snack		
Yes	460	72.1
No	178	27.9
Dinner		
Yes	552	86.5
No	86	13.5
Late dinner		
Yes	167	26.2
No	471	73.8
Mindless eating		
Yes	293	45.9
No	345	54.1
13. Food choice BEFORE the pandemic:		
Sugar-rich foods with low tooth adhesion		
Yes	281	44.0
No	357	56.0
Sugar-rich foods with high tooth adhesion		
Yes	429	67.2
No	209	32.8
High-fat foods		
Yes	376	58.9
No	262	41.1
Sausages		
Yes	321	50.3
No	317	49.7
Acidic drinks		
Yes	224	35.1
No	414	64.9
Tooth-staining foods		
Yes	445	69.7
No	193	30.3
None of them		
Yes	19	3.0
No	619	97.0
14. Food choice DURING the pandemic:		
Sugar-rich foods with low tooth adhesion		
Yes	192	30.1
No	446	69.9
Sugar-rich foods with high tooth adhesion		
Yes	429	67.2
No	209	32.8
High-fat foods		
Yes	329	51.6
No	309	48.4
Sausages		
Yes	315	49.4
No	323	50.6
Acidic drinks		
Yes	213	33.4
No	425	66.6
Tooth-staining foods		
Yes	440	69.0
No	198	31.0
None of them		
Yes	24	3.8
No	614	96.2
15. Food choice intake frequency BEFORE the pandemic:		
(1) Never	15	2.4
(2) 1–2 times a week	243	38.1
(3) 3–4 times a week	253	39.7
(4) 5–6 times a week	73	11.4
(5) 7+ times a week	54	8.5
16. Food choice intake frequency DURING the pandemic:		
(1) Never	15	2.4
(2) 1–2 times a week	156	24.5
(3) 3–4 times a week	257	40.3
(4) 5–6 times a week	122	19.1
(5) 7+ times a week	88	13.8
17. Usually, you eat the food choice selected:		
Along with main meals	56	8.8
Right after meals	115	18.0
In the morning	35	5.5
In the afternoon	262	41.1
At the night	137	21.5
Before dawn	15	2.4
Never	18	2.8
18. Tooth brushing frequency BEFORE the pandemic:		
(1) No brushing	0	0.0
(2) once a day	5	0.8
(3) twice a day	126	19.7
(4) 3 times a day	411	64.4
(5) 4+ a day	96	15.0
19. Tooth brushing frequency DURING the pandemic:		
(1) No brushing	0	0.0
(2) once a day	28	4.4
(3) twice a day	178	27.9
(4) 3 times a day	332	52.0
(5) 4+ a day	100	15.7
20. Tooth brushing time BEFORE the pandemic:		
(1) After meals and after snacks	81	12.7
(2) After meals but not after snacks	408	63.9
(3) After most meals but not all	147	23.0
(4) Not everyday	2	0.3
(5) Never	0	0.0
21. Tooth brushing time DURING the pandemic:		
(1) After meals and after snacks	104	16.3
(2) After meals but not after snacks	326	51.1
(3) After most meals but not all	196	30.7
(4) Not everyday	12	1.9
(5) Never	0	0.0
22. Health conditions BEFORE the pandemic:		
Gingivitis		
Yes	83	13.0
No	555	87.0
TMJ pain		
Yes	103	16.1
No	535	83.9
Tooth wear		
Yes	38	6.0
No	600	94.0
Cold sensitivity		
Yes	104	16.3
No	534	83.7
Aphthous ulcer		
Yes	87	13.6
No	551	86.4
Traumatic oral ulcer		
Yes	22	3.4
No	616	96.6
Herpes labialis		
Yes	9	1.4
No	629	98.6
Headache		
Yes	227	35.6
No	411	64.4
Gastroesophageal reflux		
Yes	89	13.9
No	549	86.1
None of them		
Yes	241	37.8
No	397	62.2
23. Health conditions DURING the pandemic:		
Gingivitis		
Yes	115	18.0
No	523	82.0
TMJ pain		
Yes	196	30.7
No	442	69.3
Tooth wear		
Yes	71	11.1
No	567	88.9
Cold sensitivity		
Yes	130	20.4
No	508	79.6
Aphthous ulcer		
Yes	108	16.9
No	530	83.1
Traumatic oral ulcer		
Yes	37	5.8
No	601	94.2
Herpes labialis		
Yes	14	2.2
No	624	97.8
Headache		
Yes	334	52.4
No	304	47.6
Gastroesophageal reflux		
Yes	141	22.1
No	497	77.9
None of them		
Yes	144	22.6
No	494	77.4
24. Sleep changes due to the pandemic:		
Less hours	162	25.4
No change	60	9.4
More hours at night	285	44.7
More hours at anytime (day or night)	91	14.3
More hours, almost the entire day	40	6.3
25. Parafunctional habits BEFORE the pandemic.		
Onychophagia (nail biting)		
Yes	157	24.6
No	481	75.4
Object biting		
Yes	159	24.9
No	479	75.1
Bruxism		
Awake clenching		
Yes	122	19.1
No	516	80.9
Sleep clenching		
Yes	134	21.0
No	504	79.0
Awake teeth grinding		
Yes	23	3.6
No	615	96.4
Sleep teeth grinding		
Yes	31	4.9
No	607	95.1
Dermatophagia (finger biting)		
Yes	237	37.1
No	401	62.9
Lip biting		
Yes	243	38.1
No	395	61.9
None of them		
Yes	183	28.7
No	455	71.3
26. Parafunctional habits DURING the pandemic.		
Onychophagia (nail biting)		
Yes	154	24.1
No	484	75.9
Object biting		
Yes	122	19.1
No	516	80.9
Bruxism		
Awake clenching		
Yes	197	30.9
No	441	69.1
Sleep clenching		
Yes	195	30.6
No	443	69.4
Awake teeth grinding		
Yes	49	7.7
No	589	92.3
Sleep teeth grinding		
Yes	50	7.8
No	588	92.2
Dermatophagia (finger biting)		
Yes	253	39.7
No	385	60.3
Lip biting		
Yes	279	43.7
No	359	56.3
None of them		
Yes	0	0.0
No	638	100.0
27. Alcohol consumption changes due to the pandemic:		
Never	188	29.5
Stopped	33	5.2
Decreased	173	27.1
Equal	130	20.4
Started	15	2.4
Increased	99	15.5

Table 3 shows the median, lower quartile (Q_1_) and upper quartile (Q_3_) of ordinal variables. During the pandemic, household income decreased while the perceived stress levels increased (p < 0.0001). Regarding oral hygiene, participants changed their usual tooth brushing time (p = 0.0054), and the brushing frequency decreased (p < 0.0001).

**Table 3:** Comparison of the scores for the participants’ perceived changes related to finance, stress levels, dietary habits and oral hygiene due to the Covid-19 pandemic.

Variable	Before		During		P value
	
	Median	Q_1_–Q_3_	Median	Q_1_–Q_3_	
Monthly household income	2	1–2	1	1–2	< 0.0001^a^
Stress levels	3	2–3	4	3–5	< 0.0001^a^
Food choice frequency	2	1–2	2	2–3	< 0.0001^a^
Tooth brushing frequency	4	4–4	4	3–4	< 0.0001^a^
Tooth brushing time	2	2–2	2	2–3	0.0054^a^

Q_1_: lower quartile; Q_3:_ upper quartile.

^a^ Significant at the p < 0.05 level.

During the pandemic, participants reduced breakfast (p = 0.0261), lunch (p = 0.0192), and dinner (p < 0.0001) meals from their everyday dietary habits while adding late dinner and mindless eating (p < 0.0001). As for food choices, consumption of sugar-rich foods with low tooth adhesion (p < 0.0001), fat-rich foods (p = 0.0003), and tooth-staining foods (p = 0.0489) decreased. Moreover, the frequency in choosing these types of food increased during the pandemic (p < 0.0001). Results show a poor correlation between higher stress levels and a higher food choice frequency during the pandemic (r_S_ = 0.15, p < 0.0001).

The number of participants who did not manifest signs or symptoms of health conditions decreased during the pandemic (p < 0.0001), whereas all signs or symptoms increased (p < 0.05), excepting herpes labialis (p = 0.1250). Similarly, the number of participants who did not manifest parafunctional habits before the pandemic researched zero during the pandemic (p = 0.0002). Bruxism (p < 0.0001) and lip biting (p = 0.0002) significantly increased, whereas nail and finger biting showed no significant statistical difference (p > 0.05).

Moreover, most participants noticed a change in their sleeping habits during the pandemic. Almost a quarter of the participants reported reducing their alcohol consumption. We found no correlations between sleep changes or alcohol consumption and stress levels (p > 0.05).

## DISCUSSION

As the negative consequences of the pandemic transcend physical damage^18^, we need further studies focused on correlating them to mental health and the degree of population psychological and behavioral impact^22^. The present study assessed whether the pandemic and the suspension of in-person activities affected dental students in Brazil. Given the significant increase in cross-sectional survey applications during the pandemic, we developed and validated a patient-centered questionnaire using a protocol to facilitate easy engagement. Since the construct focused on perceived changes instead of on classifying or diagnosing diseases, we rejected the null hypothesis.

A recent study suggested an increase in stress among healthcare students during the pandemic^12^, corroborating our results. Of the 638 participants, 27% reported feeling extremely stressed during the pandemic, whereas only 2% felt such stress levels before it. Moreover, 70% of the respondents claimed they felt pressured to financially contribute in their household due to decreased monthly household income. The correlations found between high stress levels, feeling pressured, and low household income suggest an association between these variables. Discontinuity of the academic routine and uncertainty regarding the graduation date may have contributed to this result, since senior students tended to feel pressured, possibly due to their suddenly postponed after-graduation plans.

Regarding dietary behavior, the frequency of main meals (breakfast, lunch, and dinner) significantly decreased while that of late dinners and mindless eating increased, finding that might be explained by changes in the students’ daily routine. Food choice frequency also increased, showing a positive correlation with high stress levels. Studies show that stress negatively affects eating behavior through the reward system in the brain. Among other conditions, stress can lead to neurobiological adaptations that promote overeating^23^.

We also observed changes in food choice. Results showed a significant decrease in the consumption of sugar-rich foods with low tooth adhesion, fat-rich foods, and tooth-staining foods. Since maintaining a balanced diet could help strengthen immunity and prevent viral infections, people opted for healthier eating habits during the pandemic^24^. Conversely, a study conducted in Italy reported an increased consumption of chocolate, ice cream, and desserts, known as comfort foods^25^. This might be explained by differences in the inclusion criteria used. While Scarmozzino and Visioli^25^ study sample included Italians at large, we specifically focused on Brazilian dental students, who may choose food based on what they know about consuming sugar-rich foods and its consequences on oral health.

Consuming sugar-rich foods reduces the pH and predisposes people to dental caries. Tooth brushing and flossing, in turn, are activities that prevent decay^26^. Surprisingly, our study found that tooth brushing frequency significantly decreased during the pandemic. A Brazilian study conducted with adults during the pandemic also found a decreased tooth brushing frequency: due to mask wearing, people were less concerned about their smiles^27^. Dental students, however, presumably know the importance of a healthy oral hygiene. Further studies are needed to understand this contradiction.

As a possible consequence of this decreased oral hygiene, all health conditions evaluated increased during the pandemic. Moreover, increased temporomandibular joint (TMJ) pain and tooth wear are concomitant with increased parafunctional habits. This finding may be associated with high-stress levels as psychological factors influence muscle hyperactivity and contribute to developing parafunctional activities^9^. These can lead to temporomandibular disorders, advanced tooth wear, and fracture of dental restorations and implants^28^.

Since the Covid-19 pandemic and its consequences could not be predicted, the present cross-sectional study is limited by the inclusion of questions regarding participants’ perceptions prior to the pandemic. Although no cause-to-effect relationship between variables can be argued, this study suggests causal hypotheses so researchers can search for correlations during the pandemic. All correlations presented here were statistically significant, thus confirming adequate power of the sample size. Concomitantly, all correlations were considered either fair or poor^29^, which puts into question the clinical relevance of these findings and implies the need for further confirmation. To achieve a more representative study sample, we applied no age restrictions to the inclusion criteria, which resulted in a mean age of 22.95 and a range from 17 to 67 years old. Having only 3.1% of the participants aged thirty years or older could be considered a limitation, since young adults would probably perceive the impacts of the pandemic differently from older adults.

Importantly, monthly household income decreased while stress levels increased among dental students during the pandemic^30^. We also observed changes in social behaviors and dietary choices, decreased oral hygiene, increased signs and symptoms of health conditions, and increased parafunctional habits. High stress levels may be associated with increased meal frequency and the pressure to contribute financially in their household. Senior students and students from reduced household income may feel more pressured.

## CONCLUSIONS

The Covid-19 pandemic significantly increased the perceived stress levels among dental students in Brazil, resulting in changes regarding dietary habits, oral health, and social-behavioral aspects.
